# Supplementary material for: Beyond treatment non-adherence: A qualitative study of clinicians’ perspectives on structural and social determinants of schizophrenia relapse in South Africa
Source: Glob Ment Health (Camb). 2026 May 5;13:e100. doi: 10.1017/gmh.2026.10200 (PMC13200024; doi:10.1017/gmh.2026.10200)
Supplement: Smit et al. supplementary material 1 — Smit et al. supplementary material [file S2054425126102003sup001.docx]

**Supplementary Table S1. Participants demographics**

| **Characteristic** | **Category** | ***n*** | **Individual years’ experience** | **%** |
| --- | --- | --- | --- | --- |
| Sex | Male | 3 |  | 21.4 |
|  | Female | 11 |  | 78.6 |
| Mean age (years) |  |  |  | 40 |
|  |  |  |  |  |
| Professional role | Psychiatrist/Psychiatric Registrar/Medical officer | 12 | #1 = 25  #2 = 8  #3 = 21  #4 = 5  #5 = 5  #8 = 7  #9 = 11  #10 =12  #11 = 9  #12 = 6  #13 = 8  #14 =13 | 85.7 |
|  | Psychiatric Nurse | 2 | #6 = 23  #7 = 21 | 14.3 |
| Highest qualification | Bachelor’s degree | 2 |  | 14.3 |
|  | Higher than bachelor’s degree | 12 |  | 85.7 |
| Work setting | Hospital (inpatient/outpatient) | 11 |  | 78.6 |
|  | Community mental health clinic | 3 |  | 21.4 |
