# Supplementary material for: Beyond treatment non-adherence: A qualitative study of clinicians’ perspectives on structural and social determinants of schizophrenia relapse in South Africa
Source: Glob Ment Health (Camb). 2026 May 5;13:e100. doi: 10.1017/gmh.2026.10200 (PMC13200024; doi:10.1017/gmh.2026.10200)
Supplement: Smit et al. supplementary material 2 — Smit et al. supplementary material [file S2054425126102003sup002.docx]

**Supplementary Table S2. Semi-structured interview guide**

**SEMI-STRUCTURED INTERVIEW GUIDE FOR FOCUS GROUP DISCUSSION WITH CLINICIANS**

**Based on your experience with relapse in schizophrenia**

- What is your understanding of relapse?
- Does treating these revolving door patients have an impact on you?
- What would you say are the main triggers of relapse?
- What limitations in the health care system do these patients face?
- How can you be empowered to help patients who relapsed?
- For those patients that are doing better, what are the strategies?
- What is your opinion on long-term patient outcomes?
- Any other thoughts or ideas related to relapse that you would like to share with us?
